# Lentiviral Gene Transfer of *Rpe65* Rescues Survival and Function of Cones in a Mouse Model of Leber Congenital Amaurosis

**DOI:** 10.1371/journal.pmed.0030347

**Published:** 2006-10-10

**Authors:** Alexis-Pierre Bemelmans, Corinne Kostic, Sylvain V Crippa, William W Hauswirth, Janis Lem, Francis L Munier, Mathias W Seeliger, Andreas Wenzel, Yvan Arsenijevic

**Affiliations:** 1 Unit of Gene Therapy and Stem Cell Biology, Jules Gonin Eye Hospital, Lausanne, Switzerland; 2 Department of Ophthalmology, University of Florida, Gainesville, Florida, United States of America; 3 Department of Ophthalmology, Program in Genetics and Tufts Center for Vision Research, Tufts University School of Medicine, Boston, Massachusetts, United States of America; 4 Unit of Clinical Oculogenetics, Jules Gonin Eye Hospital, Lausanne, Switzerland; 5 Retinal Electrodiagnostics Research Group, Department of Ophthalmology II, Eberhard-Karls University, Tübingen, Germany; 6 Laboratory of Retinal Cell Biology, University Hospital, Zürich, Switzerland; Moorfields Eye Hospital, United Kingdom

## Abstract

**Background:**

*RPE65* is specifically expressed in the retinal pigment epithelium and is essential for the recycling of 11-*cis*-retinal, the chromophore of rod and cone opsins. In humans, mutations in *RPE65* lead to Leber congenital amaurosis or early-onset retinal dystrophy, a severe form of retinitis pigmentosa. The proof of feasibility of gene therapy for RPE65 deficiency has already been established in a dog model of Leber congenital amaurosis, but rescue of the cone function, although crucial for human high-acuity vision, has never been strictly proven. In *Rpe65* knockout mice, photoreceptors show a drastically reduced light sensitivity and are subject to degeneration, the cone photoreceptors being lost at early stages of the disease. In the present study, we address the question of whether application of a lentiviral vector expressing the *Rpe65* mouse cDNA prevents cone degeneration and restores cone function in *Rpe65* knockout mice.

**Methods and Findings:**

Subretinal injection of the vector in *Rpe65*-deficient mice led to sustained expression of *Rpe65* in the retinal pigment epithelium. Electroretinogram recordings showed that *Rpe65* gene transfer restored retinal function to a near-normal pattern. We performed histological analyses using cone-specific markers and demonstrated that *Rpe65* gene transfer completely prevented cone degeneration until at least four months, an age at which almost all cones have degenerated in the untreated *Rpe65*-deficient mouse. We established an algorithm that allows prediction of the cone-rescue area as a function of transgene expression, which should be a useful tool for future clinical trials. Finally, in mice deficient for both RPE65 and rod transducin, *Rpe65* gene transfer restored cone function when applied at an early stage of the disease.

**Conclusions:**

By demonstrating that lentivirus-mediated *Rpe65* gene transfer protects and restores the function of cones in the *Rpe65* ^−/−^ mouse, this study reinforces the therapeutic value of gene therapy for RPE65 deficiencies, suggests a cone-preserving treatment for the retina, and evaluates a potentially effective viral vector for this purpose.

## Abstract 2

In the*Rpe65^-/-^* mouse model of Leber congenital amaurosis, injection of a lentiviral vector expressing the Rpe65 mouse cDNA was able to prevent cone degeneration and restore cone function.

## Editors' Summary

**Background.:**

Leber congenital amaurosis (LCA) is the name of a group of hereditary diseases that cause blindness in infants and children. Changes in any one of a number of different genes can cause the blindness, which affects vision starting at birth or soon after. The condition was first described by a German doctor, Theodore Leber, in the 19th century, hence the first part of the name; “amaurosis” is another word for blindness. Mutations in one gene called retinal pigment epithelium-specific protein, 65 kDa (RPE65)—so called because it is expressed in the pigment epithelium, a cell layer adjacent to the light-sensitive cells, and is 65 kilodaltons in size—cause about 10% of cases of LCA. The product of this gene is essential for the recycling of a substance called 11-*cis*-retinal, which is necessary for the light-sensitive rods and cones of the retina to capture light. If the gene is abnormal, the sensitivity of the retina to light is drastically reduced, but it also leads to damage to the light-sensitive cells themselves.

**Why Was This Study Done?:**

Potentially, eyes diseases such as this one could be treated by gene therapy, which works by replacing a defective gene with a normal functional one, usually by putting a copy of the normal gene into a harmless virus and injecting it into the affected tissue—in this case, the eye. The researchers here wanted to see whether expressing wild-type RPE65 using a particular type of gene vector that can carry large pieces of DNA transcript—a lentiviral vector—could prevent degeneration of cone cells and restore cone function in a mouse model of this type of LCA—mice who had had this *Rpe65* gene genetically removed.

**What Did the Researchers Do and Find?:**

Injection of the normal gene into the retina of *Rpe65*-deficient mice led to sustained expression of the protein RPE65 in the retinal pigment epithelium. Electrical recordings of the activity of the eyes in these mice showed that *Rpe65* gene transfer restored retinal function to a near-normal level. In addition, *Rpe65* gene transfer completely prevented cone degeneration until at least four months, an age at which almost all cones have degenerated in the untreated *Rpe65*-deficient mice.

**What Do These Findings Mean?:**

These findings suggest that it is theoretically possible to treat this type of blindness by gene therapy. However, because this study was done in mice, many other steps need to be taken before it will be clear whether the treatment could work in humans. These steps include a demonstration that the virus is safe in humans, and experiments to determine what dose of virus would be needed and how long the effects of the treatment would last. Another question is whether it would be necessary (or even possible) to treat affected children during early childhood or when children start losing vision.

**Additional Information.:**

Please access these Web sites via the online version of this summary at http://dx.doi.org/10.1371/journal.pmed.0030347.

- The Foundation for Retinal Research has detailed information on Leber's congenital amaurosis
- Contact a Family is a UK organization that aims to put families of children with illnesses in touch with each other
- The Foundation for Fighting Blindness funds research into, and provides information about many types of blindness, including Leber's congenital amaurosis
- This Web site provides information on gene therapy clinical trials, including those dedicated to cure eye diseases
- This foundation provides information on diseases leading to blindness, including Leber's congenital amaurosis

## Introduction

The neuroretina is composed of several cell layers in which light stimuli are converted into an electric signal by the photoreceptors. This conversion process takes place in the visual pigment of the photoreceptors, which is composed of opsin, a protein component, and 11-*cis*-retinal, a vitamin A chromophore. Absorption of light triggers isomerisation of the chromophore into all-*trans*-retinal and conformational change of the opsin. Upon this activation, the phototransduction cascade is initiated, which finally results in the generation of the electric signal. To regain light sensitivity, all-*trans*-retinal must be re-isomerised to 11-*cis*-retinal. This regeneration requires transport of the all-*trans*-retinal to the adjacent retinal pigment epithelium (RPE), where a number of enzymatic reactions are involved in the re-isomerisation to 11-*cis*-retinal, before transport of the chromophore back to the photoreceptors [1]. The pigment epithelial protein RPE65 is essential for this process [2] because it catalyses the re-isomerisation of the all-*trans*-retinoid into the 11-*cis* form [3–5]. In agreement with this essential function, mice deficient for RPE65 have strongly reduced retinal function [2,6]. In humans, *RPE65* mutations cause a form of early-onset retinal dystrophy termed Leber congenital amaurosis [7–9], which leads to early blindness.

The ability to efficiently target RPE cells with viral vectors has led to the proposal that RPE65 deficiencies are good candidates for gene therapy protocols [10,11]. In 2001, a study by Acland et al. [12] revealed the clinical potential of *Rpe65* gene transfer. In this study, the treatment of RPE65-deficient dogs by intraocular delivery of a recombinant adeno-associated virus (rAAV) encoding canine *Rpe65* partially rescued retinal function and restored vision. Furthermore, long-term follow-up of the treated dogs showed that the visual recovery was stable for more than three years without adverse effects [13]. A second study, by Narfström et al. in 2003 [14], using the same animal model treated with a rAAV-encoding canine *Rpe65* also demonstrated a long-term improvement of the visual function up to three years postinjection, associated with a clear improvement in a behavioural tests conducted in both day and night vision [14,15]. As a consequence of these results, two clinical trials involving rAAV-mediated *RPE65* gene transfer were proposed in 2004 in the US, the first being accepted while the second is still under review (NIH GeMCRIS, http://www.gemcris.od.nih.gov/ protocol numbers 0410–677 and 0510–740).

It is important, however, to continue to document the effectiveness of *RPE65* gene transfer in animal models, especially in light of the results obtained recently after rAAV-mediated gene transfer of *RPE65* in the *Rpe65* knockout mouse *(Rpe65* ^−/−^ *):* Gene transfer was effective when administered in utero, but treatment of adult mice led to only limited improvement of retinal function [16,17]. Therefore, the timing of gene therapy seems to be crucial for the outcome. This is particularly true when aiming at cone vision rescue, which is of prime importance for human patients. Interestingly, cone function is lost in *Rpe65* ^−/−^ mice [6] due to early-onset degeneration of these cells [18,19], and Chen et al. [20] demonstrated recently that adenoviral delivery of *Rpe65* in *Rpe65* ^−−/−^ mice restores isomerohydrolase activity and prevents, to a certain extent, early cone loss, although this latter study did not examine the cone function. rAAV-mediated *Rpe65* gene transfer can also moderately increase cone cell survival, but its effect on the function of these cells remains unclear [17]. Thus, RPE65 seems essential for cones, but the effect of *Rpe65* gene transfer on their survival and function remains to be elucidated.

Most of the work published to date in the field of *RPE65* gene transfer involved the use of rAAV vectors. These vectors efficiently target retinal cells, and in particular RPE and photoreceptors when they are injected in the subretinal space [21–23]. Moreover, the use of different viral serotypes allows the targeting of both types of cells or the RPE alone [10]. The cloning capacity of rAAV vectors is, however, limited to 4 kb, which hampers the use of large transgenes—for instance *ABCA4,* the gene mutated in Stargardt disease. In contrast, lentiviral vectors appear as an attractive alternative to rAAV for gene therapy protocols targeting RPE cells: The lentiviral vector has a cloning capacity of up to 10 kb, causes a weak or absent inflammatory response, and expresses transgenes rapidly. Most importantly, the lentiviral vector transduces RPE cells with high efficiency [10,24–27], making of it an ideal candidate for *Rpe65* gene transfer.

For the present study, we used *Rpe65* ^−/−^ mice to analyze the therapeutic effect of lentivirus-mediated gene transfer on survival and function of the affected retinal cells. As cone impairment and degeneration progress rapidly in *Rpe65* ^−/−^ mice, we performed a detailed analysis of the therapeutic effect on this photoreceptor subtype. We also used a double-knockout mouse strain modelling RPE65 deficiency on a pure cone function background to study the effect of gene transfer on cone function.

## Methods

### Construction and Production of Lentiviral Vectors

The lentiviral plasmids used in his study were derived from the pLOX-GFP plasmid [28], which contains the central polypurine tract and central termination sequence (cPPT/CTS [29]) and the woodchuck hepatitis virus post-transcriptional regulatory element (WPRE [30]) downstream of the transgene. To construct the control vector encoding GFP, the PGK-GFP expression cassette was excised from the pLOX-GFP plasmid using ClaI (blunted) and BamHI sites, and replaced by the R0.8-GFP cassette previously extracted by KpnI (blunted) and BamHI digestion from the pRPE0.8-GFP plasmid [13,31]. The plasmid encoding *Rpe65* was then generated by insertion of RPE65 mouse cDNA in place of the GFP ORF in the control plasmid using BamHI and XhoI sites.

Recombinant lentiviral particles were produced by transient transfection of 293T cells as previously described [27,28]. Viral supernatants were concentrated by ultracentrifugation at 70,000 *g* for 90 min at 4 °C. Finally, to achieve a 1,000-fold concentration of the initial supernatant, viral pellets were resuspended in a minimal of volume of PBS containing 10 mg/ml BSA. Aliquots of 5–10 μl were then stored at −70 °C until further use. Total particle concentration of the viral stocks was estimated by quantification of the p24 capsid protein using RETRO-TEK HIV-1 p24 Antigen ELISA kit (ZeptoMetrix, Buffalo, New York, United States) according to the manufacturer's instructions. In addition, infectious titres of the Hlox-R0.8-GFP vector were quantified by infection of 293T cells, in which the R0.8 promoter is active. Briefly, 293T cells were seeded on 12-well plates and infected by serial dilutions of the vector stocks. On day 4 postinfection, cells were harvested, dissociated, and fixed for 15 min in 4% paraformaldehyde in PBS. The infectious titres were deduced from the percentage of GFP-expressing cells estimated by flow cytometry on a FACSCalibur (Becton Dickinson, Franklin Lakes, New Jersey, United States). Infectious titres were always in the range of 3 transducing units per pg of p24 protein.

### Animals and Surgical Procedures

Animals were handled in accordance with the statement of the “Animals in Research Committee” of the Association for Research in Vision and Ophthalmology (Rockville, Maryland, United States), and protocols were approved by the local institutional committee (Service vétérinaire du canton de Vaud, Lausanne, Switzerland). Mice were kept at 22 °C under a 12 h light–12 h dark cycle with lights on at 7 a.m., and were fed ad libitum. *Rpe65* ^−/−^ mice were obtained from Dr. T. M. Redmond [2], and *Rpe65* ^−/−^/*Gnat1a* ^−/−^ double knockout mice were described previously [32].

Before administration of lentiviral vectors, mice were transferred to a level 2 confinement room. For intravitreal injection at postnatal day 5 (P5), mouse pups were anaesthetized by volatile anaesthesia (2%–3% isoflurane with O_2_ at a rate of 800 ml/min). A horizontal incision was made on the superior eyelid to access the eye. A 34-gauge bevelled needle mounted on a 5 μl syringe (Hamilton, Bonaduz, Switzerland) was introduced in the vitreal cavity through the dorsal pole of the sclera, without touching the lens. 1 μl of viral suspension diluted in PBS containing 1% BSA was injected into the vitreous, and the needle was left in place for additional 30 s. Mouse pups were then returned to their mothers after they regained consciousness. For subretinal injection during adulthood, the mice were anesthetized by volatile anaesthesia (1.5%–3% isoflurane with O_2_ at a rate of 800 ml/min). The superior eyelid was maintained open by traction using a suture wire. The eye globe was then immobilized by two suture wires inserted respectively in nasal and temporal positions in the conjunctiva at the proximity of the limb. Using fine ophthalmic scissors, the conjunctiva of the dorsal pole was dissected, and the sclera/choroid layers were punctured with an ophthalmic lancet. A 34-gauge bevelled needle mounted on a 5 μl syringe was then tangentially inserted into this hole to reach the posterior temporal part of the subretinal space. 1.5 μl of viral suspension was injected to create a subretinal bleb, and the needle was left in place 30 s before withdrawal.

### ERG

ERG recordings were performed on site at Jules Gonin Eye Hospital (Lausanne, Switzerland). For scotopic conditions, mice were dark-adapted overnight. Subsequent procedures were undertaken under dim red light unless otherwise specified. Mice were anaesthetized with a mixture of ketamine (100 mg/kg) and xylazine (15 mg/kg), and pupils were dilated by topical administration of 0.5% tropicamid (Novartis Pharma, Bern, Switzerland). Mice were then positioned on a heated platform to prevent a drop in body temperature. A reference electrode and a ground electrode adapted from 30-gauge needles were inserted below the skin of, respectively, the scalp and the back of the mouse. Recording electrodes made of thin silver wires were put in contact with the surface of the cornea, which was then covered with a carboxymethylcellulose sodium solution (Celluvisc, Allergan, Irvine, California, United States) to prevent drying of the cornea and to ensure maximal contact with the electrodes. The mouse was placed in the centre of a Ganzfeld stimulator from a Multiliner Vision apparatus (Jaeger/Toennies, Höchberg, Germany) adapted for examination of rodents, and placement of the electrodes was corrected until the impedance level dropped below 10 kOhm. The corneal ERG was then recorded in scotopic conditions in response to single flashes of white light of the following intensities: 1 × 10^−^^4^, 1 × 10^−^^3^, 1 × 10^−^^2^, 3 × 10^−^^2^, 1 × 10^−^^1^, 3 × 10^−^^1^, 1, 3, 10, and 25 candelas (cd) s/m^2^, generated by a stroboscopic light located in the upper part of the Ganzfeld stimulator. For the photopic ERG, the mice were left in the Ganzfeld stimulator where they were adapted for 10 min to a white light of 30 cd/m^2^. Photopic responses were then recorded in response to single flashes ranging from 10−^2^ to 25 cd s/m^2^, in the same illumination conditions. For each stimulus intensity, ten to 15 traces were averaged. Amplitude of the a-wave was defined as the difference between the baseline level at the time of stimulation and the peak of the a-wave. Amplitude of the b-wave was defined as the difference between the peak of the b-wave and the peak of the a-wave (or the baseline level when the a-wave was not detectable). Amplitudes are expressed in μV. At the end of the examination, the eyes were covered with Viscotears (Novartis Pharma), and the mice were kept under surveillance until they regained consciousness.

### Histology

#### Tissue processing.

After mice were sacrificed, the eyes were enucleated, a hole was created through the cornea, and the eyes were fixed for 1 h in 4% paraformaldehyde in PBS. After overnight incubation in 25% sucrose, the eyes were embedded in albumin from hen egg white (Fluka, Buchs, Switzerland) and cut in 14 μm sections using a cryostat. Sections were collected on six serial slides for each eye allowing multiple labelling throughout the entire eye for each slide.

#### Immunolabelling.

For immunohistological analysis, the slides were blocked for at least 1 h at room temperature in PBS with 10% normal goat serum (Dako, Zug, Switzerland) and 0.2% Triton X-100. Most of the time the primary and secondary antibodies were incubated in PBS with 2% normal goat serum and 0.2% Triton. Mouse monoclonal anti-RPE65 (Abcam, Cambridge, United Kingdom) was incubated overnight at room temperature at 1:1,000, while rabbit polyclonal anti-RPE65 (pin5 [33]) was incubated overnight at 4 °C at 1:500. Rabbit polyclonal anti-GFP (Abcam) was incubated overnight at room temperature at 1:2,000. Peanut agglutinin lectin (PNA) labelling was performed using PNA linked to TRITC (Sigma, Buchs, Switzerland) diluted at 1:1,000 and incubated overnight at 4 °C. Rabbit polyclonal anti-GNAT2 (Santa Cruz Biochemicals, Santa Cruz, California, United States) was incubated overnight at room temperature at 1:100, anti-S-opsin (Santa Cruz Biochemicals) and anti-MWL (Chemicon, Temecula, California, United States) overnight at 4 °C at 1:1,000. Secondary antibodies were goat anti-rabbit linked to FITC (1:100, Jackson Immunoresearch, Westgrove, Pennsylvania, United States) or Cy3 (1:500, Jackson Immunoresearch) or goat anti-mouse linked to AF488 (1:500, Molecular Probes, Eugene, Oregon, United States) were incubated 1 h at room temperature. For immunolabelling using the anti-S-opsin, horse serum (Jackson Immunoresearch) and anti-goat-Cy3 (1:1,000; Jackson Immunoresearch) were used. Before examination, sections were counterstained with DAPI (Molecular Probes) and mounted under coverslips with Mowiol 4–88 Reagent (VWR International AG, Lucerne, Switzerland).

#### Quantification.

Sections were analyzed on a BX60 microscope equipped for epifluorescence (Olympus Suisse SA, Aigle, Switzerland) and coupled to the analySIS 3.0 software (Soft Imaging System, Munster, Germany). For quantification of the transduced area, images of the whole area expressing the transgenes were digitized on serial sections separated by 84 μm, the length of the transduced part of the RPE was calculated with the analySIS software, and the area between each section was deduced by multiplication of this length by the intersection distance. To quantify the number of cells positive for PNA, GNAT2, and M/L- and S-opsin, we chose for each eye the section that cut the optic nerve in the middle and was thus the most central. We then counted throughout the retina of this section the number of outer segments that stained positive.

### Statistical Analysis

Statistical analyses of the ERGs were done using StatView 5.0 software. Histological counting of cone markers was analyzed by one-way ANOVA to determine the statistical significance between the different groups (LV-RPE65 treated, LV-GFP treated, *Rpe65* ^−/−^, and wild-type mice).

## Results

### LV-RPE65 Allows Efficient and Specific RPE65 Protein Expression in the Retinal Pigment Epithelium of *Rpe65* ^−/−^ Mice

A major concern in gene therapy protocols is to avoid expression of the therapeutic transgene to nontarget organs and cells. The *Rpe65* gene is expressed mainly in the RPE. To limit expression of the transgene to this site, we constructed an HIV-1-derived lentiviral vector known to transduce the RPE with high efficiency when delivered in the subretinal space and with limited diffusion toward the other retinal layers [10,24–27]. Furthermore, we used a 0.8 kbp proximal fragment of the human *RPE65* gene (R0.8) with the aim of driving transgene expression specifically in RPE cells.

**Figure 1:**
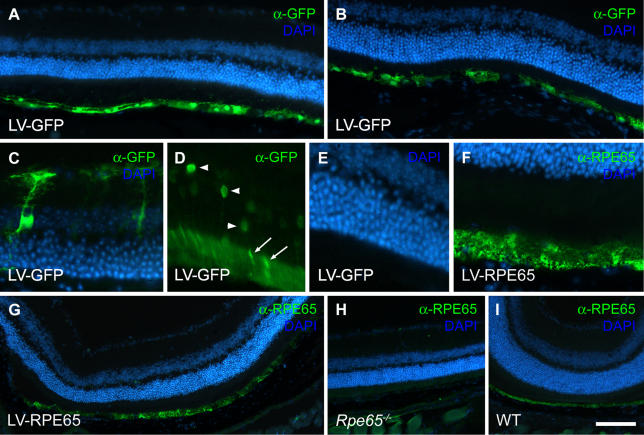
The Lentiviral Backbone Containing the R0.8 Promoter Fragment Efficiently Expresses a Transgene in the Mouse RPE *Rpe65* ^−/−^ mice received the LV-GFP vector (A–D) or the LV-RPE65 vector (F and G) as an intravitreal injection at P5 (A, D–G) or a subretinal injection at 1 mo of age (B and C). 1 wk later GFP expression in the RPE was detected spanning a large proportion of the retina (A and B). Although to a lesser extent, transduced cells were also evident in the photoreceptor layer after injection at P5 (D). GFP expression was detected in the ONL (arrowheads in D) as well as in the outer segments (arrows in D). (E) DAPI counterstaining of the sample in (D). GFP was also detected occasionally in Müller cells after injection in the subretinal space at the age of 1 mo (C). After injection of the LV-RPE65 vector, immunolabelling showed RPE65 expression in the RPE (F and G). (H and I) Control RPE65 immunolabelling on noninjected *Rpe65* ^−/−^ retina (G) and wild-type retina (H). Scale bar in (I): 100 μm in A, B, and G–I; 25 μm in C–F.

In a first series of experiments we characterized the pattern of transgene expression from the R0.8 promoter in the lentiviral context (Figure 1). We treated *Rpe65* ^−/−^ mice by intravitreal injection at P5 or subretinal injection at 1 mo with HIV-1-derived lentiviral vectors encoding either the GFP reporter gene (LV-GFP) or the *Rpe65* cDNA (LV-RPE65), under the control of the R0.8 promoter. In both cases, immunofluorescence labelling performed 1 wk after gene transfer revealed GFP (Figure 1A–1D) or RPE65 protein expression (Figure 1F and 1G) in a large area of the RPE. In addition, immunolabelling of GFP led to detection of other cell types in the retina. In some cases, a few photoreceptors were transduced after intravitreal injection of neonates only (Figure 1D), as well as occasionally some Müller cells in subretinal injection of adults (Figure 1C). In the latter case, the transduction of Müller cells seemed to be limited to a lesion of the retina due to the surgical procedure. These ectopic expressions always accounted for a very low percentage of the transduced cells. After LV-RPE65 injections, the presence of RPE65 protein in the RPE was confirmed by the use of two different antibodies (see Materials and Methods). Nevertheless, contrary to what we observed after GFP gene transfer, we did not detect RPE65 protein in either photoreceptors or Müller cells. This most probably reflects the difference in detection sensitivity between the GFP and the RPE65 antibodies. These results demonstrate the usefulness of the lentiviral vector and the R0.8 promoter to re-express the RPE65 protein in the RPE of RPE65-deficient mice.

Next we undertook a quantitative study to characterize transgene expression in the RPE at a later time point. *Rpe65* ^−/−^ mice at age 5 d received a bilateral injection in the vitreous of LV-GFP (*n* = 5) or LV-RPE65 (*n* = 5) at a dose of 20 ng of p24 (corresponding to 6 × 10^4^ transducing units for the GFP vector), and transgenes expression was analyzed at the age of 4 mo. Cryostat serial sections were immunolabelled, and the extent of RPE stained for either GFP or RPE65 was determined. We used the intersection distance to deduce the total area of the RPE expressing the respective transgenes. This quantification revealed long-lasting transgene expression. We determined a transduced area of 0.93 ± 0.33 mm^2^ in the LV-GFP-treated group and of 0.32 ± 0.2 mm^2^ in the LV-RPE65-treated group. We also observed significant heterogeneity in the extent of the transduced area within each group. Some eyes displayed transgene expression spanning a large area of the retina, whereas others showed only little or no expression. This heterogeneity was most probably due to the surgical procedure, which, in our hands, remains highly variable. In summary, the histological analysis demonstrated long-term (4 mo) expression of the transgenes in areas of variable size.

### *Rpe65* Gene Transfer at P5 Restores Retinal Function of *Rpe65* ^−/−^ Mice

**Figure 2:**
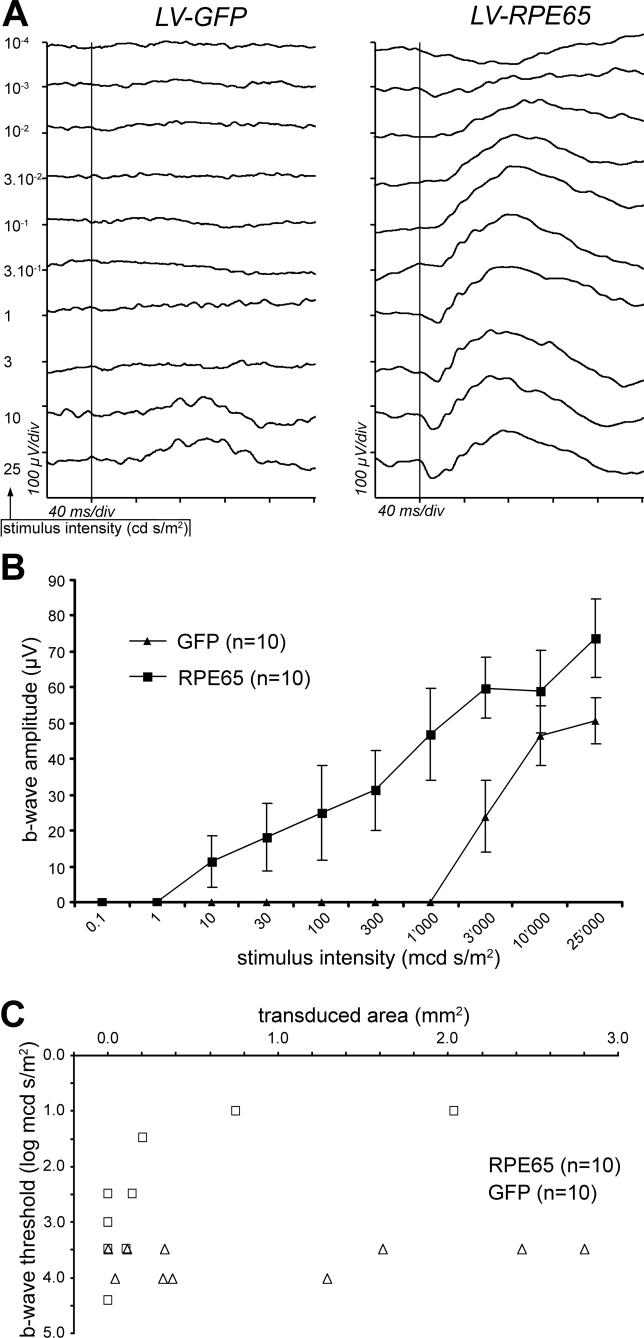
The LV-RPE65 Vector Restores the ERG Response of the *Rpe65* ^−/−^ Mice *Rpe65* ^−/−^ mice were treated P5 by an intravitreal injection of 20 ng of lentiviral vector. (A) ERG response to single flashes was recorded in scotopic condition at 2 mo of age. After LV-GFP treatment, the mice showed a response typical for *Rpe65* ^−/−^ mice, with the a-wave absent and the b-wave elicited only for the highest stimuli (i.e., 10 and 25 cd s/m^2^; left tracings). After LV-RPE65 treatment, a stimulus of 10^−^^2^ cd s/m^2^ was sufficient to trigger the b-wave, and the a-wave appeared for a stimulus of 0.3 cd s/m^2^ (right tracings). Vertical bars indicate occurrence of the stimuli; x-axis: 40 ms/div; y-axis: 100 μV/div. (B) Summary of the ERG recordings performed 2 mo after vector delivery at P5 in *Rpe65* ^−/−^ mice. Amplitude of b-wave (expressed in μV) is represented for each stimulus intensity. In the LV-RPE65-treated group (squares), the b-wave rose with a stimulus as low as 10^−^^2^ cd s/m^2^, whereas in the LV-GFP-treated group (triangles) 3 cd s/m^2^ were necessary to elicit the b-wave. ANOVA for repeated measures showed significant stimulus intensity effect (*p* < 0.0001), group effect (*p* = 0.023), and group versus stimulus intensity interaction (*p* = 0.002). Data are presented as mean ± standard error of the mean (SEM). (C) Improvement of the ERG response after LV-RPE65 treatment is correlated with the level of RPE65 expression. The area of the retina expressing GFP (triangles) or RPE65 (squares) was quantified for each eye at age 4 mo after gene transfer at P5, and plotted against the b-wave threshold (defined as the intensity of the lowest stimulus capable of eliciting the b-wave, expressed in log mcd s/m^2^).

To reveal whether *Rpe65* transgene expression results in an improvement of retinal function, we recorded the ERG response in *Rpe65* ^−/−^ animals 2 mo following vector injection at P5. In uninjected *Rpe65* ^−/−^ mice, the ERG recorded in scotopic conditions was characterized by an increase of the response threshold (i.e., the retina is less sensitive to light), when compared to wild-type animals. In line with previous studies [6], a 3.5 log unit (about 3,000-fold) lower stimulus was indeed sufficient to elicit in wild-type animals a b-wave of similar amplitude to the one observed for a stimulus of 3 cd s/m^2^ in *Rpe65* ^−/−^ mice. Furthermore, in uninjected *Rpe65* ^−/−^ mice, we never detected an a-wave (unpublished data). Similarly, in the LV-GFP treated group, the threshold of the b-wave appeared for the most intense stimuli, i.e., 3–10 cd s/m^2^, and the a-wave was never detected (Figure 2A, left tracings). In contrast, the LV-RPE65 treatment improved to a great extent the b-wave threshold of the *Rpe65* ^−/−^ mice (Figure 2A, right tracings). In this group, the threshold was improved for 6/10 eyes and, in the best cases (*n* = 2), the b-wave response appeared for stimuli as low as 10^−^^2^ cd s/m^2^ (i.e., 10-fold less sensitivity than wild type, compared to the 3,000-fold less sensitivity of the LV-GFP group) and could reach an amplitude equivalent to 34.6% of the mean of wild-type controls (333 ± 32.6 μV; *n* = 12) at the highest stimulus intensity (25 cd s/m^2^). As did the b-wave, an a-wave was clearly evidenced in the animals showing the best responses, which reached 23.1% of the wild type mean (128.9 ± 15.3 μV; *n* = 12) for the 25 cd s/m^2^ stimulus. Although the restoration of retinal function after LV-RPE65 treatment was variable, the statistical analysis of the b-wave amplitude showed a significant difference between LV-GFP and LV-RPE65 treatments (Figure 2B, *p* = 0.023 determined by ANOVA for repeated measures). It is important to note that we have included in this statistical analysis all the treated animals without discrimination, i.e., even those for which transgene expression was low or undetectable (see quantification below).

To assess whether the degree of ERG restoration was correlated with the size of the RPE area expressing *Rpe65* after gene transfer, we attributed a functional score to each eye. This score was defined as the lowest stimulus intensity (expressed in log mcd s/m^2^) for which a rise of the b-wave was evident (response threshold), and was plotted as a function of the area of RPE expressing the transgene (GFP or *Rpe65;* Figure 2C). In the LV-GFP group, no b-wave was triggered by stimuli below 3.5 log mcd s/m^2^ regardless of the size of the transduced area (Figure 2C). In contrast, a b-wave was elicited in some mice of the LV-RPE65 group by stimuli as low as 1 log mcd s/m^2^, the mice displaying the better sensitivity being also those with the largest transduced area (Figure 2C). This confirmed that LV-RPE65 treatment restores retinal function and that the degree of restoration depends on the size of the transduced area.

### LV-RPE65 Treatment Delays Cone Degeneration in *Rpe65* ^−/−^ Mice

**Figure 3:**
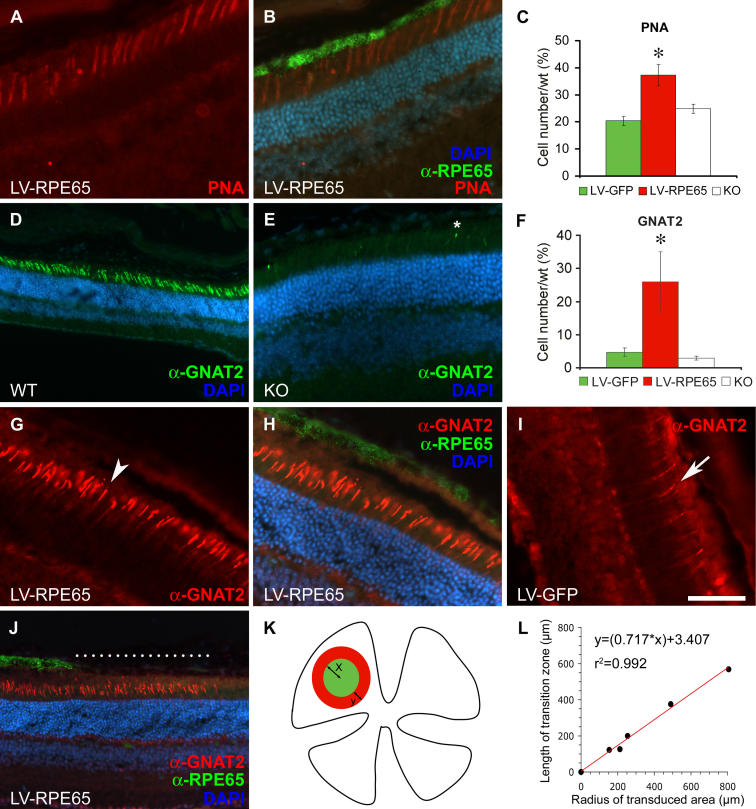
PNA and GNAT2 Labelling Increase after LV-RPE65 Treatment at P5 of *Rpe65* ^−/−^ Mice (A and B) PNA linked to TRITC labels cone outer segments in red in a LV-RPE65-treated eye (A). (B) Merged picture representing RPE65 (green), PNA labelling (red), and cell nuclei (blue) in the same section used in (A). (C) PNA labelling was quantified by counting the number of PNA-positive outer segments in the sagittal section that bisected the optic nerve for each eye of LV-RPE65 (*n* = 9) or LV-GFP (*n* = 7) treated groups as well as of *Rpe65* ^−/−^ (KO; *n* = 4) and wild-type control groups (*n* = 4). Values represent percentage of wild type (set to 100%). A significant increase of PNA labelling was seen for LV-RPE65 treated group compared to the LV-GFP (*n* = 7, *p* = 0.007) or untreated (*n* = 4, *p* = 0.018) groups (*p < 0.05). Data are presented as a mean percentage of the wild-type value ± SEM. (D) GNAT2 (revealed in green by a secondary antibody linked to FITC) in a wild-type retina. (E) The GNAT2 staining is extremely weak in *Rpe65* ^−/−^ mice at age 4 mo. *Example of remaining outer segment staining counted in the *Rpe65* ^−/−^ and LV-GFP treated groups. (F) Quantification of GNAT2 labelling was performed (as described for PNA labelling) and showed a significant increase of GNAT2 expression in the LV-RPE65 group (*n* = 6) compared to the LV-GFP (*n* = 8, *p* = 0.017) or untreated (*n* = 4, *p* = 0.03) groups (*p < 0.05). The discrepancy in cone loss between PNA and GNAT2 markers in *Rpe65* ^−/−^ (compare control groups in C and F) is most probably due to the persistence of the cone interphotoreceptor matrix sheath, which can still be present after other cone markers have disappeared [42]. Data are presented as a mean percentage of the wild-type value ± SEM. (G and H) A GNAT2 pattern similar to that observed in wild-type mice is recovered after LV-RPE65 treatment in *Rpe65* ^−/−^ mice (G). Double labelling shows GNAT2 stained in red, RPE65 in green (H). Example of outer segment positive for GNAT2 is pointed by an arrowhead. (I) LV-GFP treatment does not improve GNAT2 expression in *Rpe65* ^−/−^. Example of weak residual labelling for GNAT2 in a control eye treated with LV-GFP (arrow). (J) After LV-RPE65 treatment, a transitional area with spared GNAT2-positive cones but devoid of RPE65 expression was observed (dashed line). (K and L) In (K) the diagram depicts a transitional area in a ring (red) surrounding the transduced area (green). The radius of the transduced area is indicated by x; the width of the transitional area is indicated by y. The relation of x to y is graphed in (L); the radius of the transduced area is linearly correlated with the transitional area. Scale bar in (I): 50 μm in A, B, E, and G–I; 100 μm in D and J. KO, *Rpe65* ^−/−^ mice; LV-GFP, LV-GFP-treated mice; LV-RPE65, LV-RPE65-treated mice; WT, *Rpe65* ^+^^/^^+^ mice.

Previous studies have described cone loss of function and degeneration in *Rpe65* ^−/−^ mice as early as at 2 wk of age [6,17,19,34]. However, cone survival after *Rpe65* gene transfer has not been extensively investigated so far [16,17]. We therefore analyzed in detail cone morphology and the expression of cone-specific markers by quantifying the expression of different markers at 4 mo of age on sections adjacent to the one used for quantification of GFP and *Rpe65* expression (after gene transfer at P5, see above). We first labelled the retinas with PNA, which binds the interphotoreceptor matrix sheath surrounding cone outer segments (Figure 3A and 3B). For each eye, PNA-positive structures were counted across the entire span of the retina in sections that cut through the optic nerve head. The optic nerve was used here as a reference to select a central section on which to quantify the different markers. This retinal area does not necessarily correspond to the site of maximal transgene expression, but, considering that cone markers are subjected to regional variability, it has the advantage of delimiting a reproducible area for cell counting. We observed a significantly higher number of PNA-labelled structures after LV-RPE65 treatment (258 ± 24, *n* = 9) compared to LV-GFP treatment (141 ± 10, *n* = 7, *p* = 0.007) or untreated mice (172 ± 10, *n* = 4, *p* = 0.018). Moreover, the best scores were obtained in eyes that most widely expressed the *Rpe65* transgene as evaluated by the total area of RPE expressing RPE65 protein (unpublished data) and reached up to 50% (mean 37% ± 4%, *n* = 9) of the amount of PNA present in the wild-type control (Figure 3C). This quantification demonstrated that LV-RPE65 treatment at P5 conserves the interphotoreceptor matrix sheath of cones for an extended period of time.

To assess cone survival more specifically, we used immunofluorescence labelling of the cone-specific protein transducin (GNAT2), the mRNA level of which is drastically reduced at 2 mo of age in *Rpe65* ^−/−^ mice [19]. In wild-type animals, GNAT2 immunolabelling revealed numerous cone outer segments (Figure 3D), while almost no GNAT2 immunoreactivity was detectable in the whole retina of *Rpe65* ^−/−^ mice at 4 mo of age (Figure 3E, asterisk). Treatment of *Rpe65* ^−/−^ mice with LV-RPE65, but not with LV-GFP, significantly increased the number of outer segment labelled with GNAT2 (Figure 3F–3I). Similar to the results obtained for PNA affinity, the best rescue of GNAT2 labelling was observed in eyes with widespread *Rpe65* expression (unpublished data) and reached up to 50% (mean 26% ± 9%, *n* = 6) of the amount of GNAT2 present in the wild-type control. This protective effect of *Rpe65* gene transfer corresponds to a 5-fold increase in correctly localized GNAT2 labelling compared to untreated mice (Figure 3F).

All these results represent a mean for the entire section cutting the optic nerve in the middle, but do not accurately show regional rescue at the site of transgene expression. To address this point, we counted the number of GNAT2-positive outer segments in the RPE region expressing the *Rpe65* transgene on sections double-labelled for GNAT2 and RPE65 (Figure 3H), and thus established a cone density expressed as cell number per millimeter of epithelium. We found similar numbers of cones labelled for GNAT2 per millimeter for both LV-RPE65 (154 ± 29 cells/mm, *n* = 4) and wild-type controls (143 ± 12 cells/mm, *n* = 4). Finally, we noted that the area of GNAT2 staining (i.e., cone rescue) was larger than the area with detectable RPE65 expression. We measured the length of the transitional area (the area with GNAT2-positive cells but without RPE65 expression, Figure 3J, dashed line). Assuming that the transduced area is circular (Figure 3K), we determined its radius (“x” in Figure 3K) and found a linear correlation (Figure 3L) with the width of transitional area (“y” in Figure 3K). In summary, injection of the LV-RPE65 vector at P5 leads to correct GNAT2 expression in *Rpe65* ^−/−^ mice even 4 mo after the treatment not only at the site of transgene expression but in the surrounding area as well.

**Figure 4:**
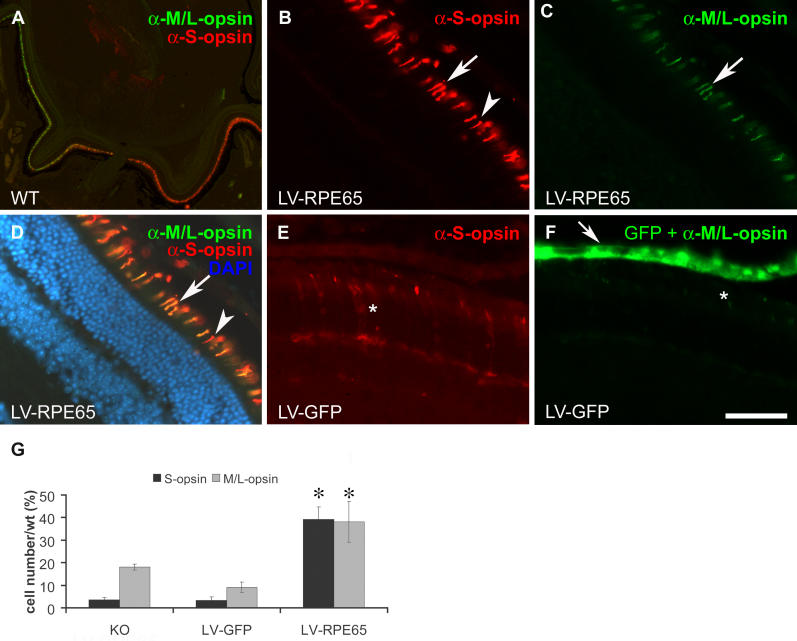
S- and M/L-Cone Opsin Labelling Increased after LV-RPE65 Treatment of *Rpe65* ^−/−^ Mice at P5 (A) Double immunostaining of wild-type retina with antibodies directed against S-opsin (red) and M/L-opsin (green) localized in the outer segment. Note that some cones express both opsins while others are mainly labelled for only one of these opsins. (B–D) LV-RPE65 treatment allows similar expression of both S- and M/L-opsin to the wild-type retina in the region expressing the *Rpe65* transgene. Arrows indicate double labelling of cone opsins; arrowheads indicate cone stained only for S-opsin. (E and F) LV-GFP treatment does not rescue S- or M/L-opsin expression and shows a pattern of staining similar to untreated *Rpe65* ^−/−^ (unpublished data). *Mislocalized and reduced S- and M/L-opsin expression. (G) Quantification of the three eyes with the highest transgene expression for LV-RPE65 and LV-GFP groups shows a significant increase in both S- and M/L-opsin expression after LV-RPE65 treatment compared to the LV-GFP (*p* = 0.005 for M/L-opsin and *p* < 0.0001 for S-opsin) or untreated (*n* = 4, *p* = 0.024 for M/L-opsin and *p* < 0.0001 for S-opsin) groups (*p < 0.05). No statistical difference was noted between LV-GFP and untreated knockout animals. Data are presented as mean ± SEM. Scale bar in (F): 500 μm in A; 50 μm in B–F. KO, *Rpe65* ^−/−^ mice; LV-GFP, LV-GFP-treated mice; LV-RPE65, LV-RPE65-treated mice; WT, *Rpe65* ^+/+^ mice.

Two other cone markers that are important for cone function are S- and M/L-opsin. Both of these opsins are thought to be expressed in different proportions in the same cell, thus determining the precise photoreceptor type of the cell, i.e., blue cone (predominant S-opsin expression) or red/green cone (predominant M/L-opsin expression) [35]. It has been shown that expression of these opsins is reduced and that the remaining proteins are mislocalized in *Rpe65* ^−/−^/*Rho* ^−/−^ double knockout mice as early as P25, identifying 11-*cis*-retinal as a key partner for correct cone opsin trafficking [18]. In light of this information, we analysed the effect of LV-RPE65 treatment on the amount and distribution of the two cone opsins (Figure 4). In the wild-type retina, the S-opsin and M/L-opsin antibodies strongly labelled the outer segments of different or common subsets of cones with a gradient of expression leading to a predominantly dorsal expression of the M/L-opsin and a predominantly ventral expression of the S-opsin (Figure 4A). In *Rpe65* ^−/−^ mice, a marked decrease in both the M/L-opsin and the S-opsin was noticed with rare labelling showing mostly mislocalized proteins (unpublished data). Similarly, in LV-GFP-treated mice, the rare and weak staining present at this age was mostly mislocalized in the cellular body (Figure 4E and 4F). After LV-RPE65 treatment, we observed an increase in expression of both opsins, which had adopted the correct cellular localization in the outer segments (Figure 4B and 4D). We compared the three eyes showing maximal transgene expression for each group (as determined by the RPE layer area expressing GFP or RPE65) and obtained a significant improvement of both S- and M/L-opsin expression in LV-RPE65-treated animals (respectively, on average 39% ± 5% and 38% ± 9% of the wild-type amount) compared to LV-GFP treated (respectively, on average 3% ± 1.5% and 9% ± 2.3% of the wild-type amount) or untreated knockout (respectively, on average 4% ± 1% and 18% ± 1.4% of the wild-type amount) (Figure 4G). Using the same methodology as for GNAT2 staining, we also quantified the extent of the transitional area on sections double labelled for RPE65 and S-opsin (*n* = 5). Similar to the quantification of GNAT2 transitional area depicted in Figure 3L, we found a strong correlation between the radius of the transduced area and the extent of the transitional area quantified on S-opsin-stained sections (y = 0.736x + 16.77; *r* ^2^ = 0.988).

In summary, we show that 4 mo after LV-RPE65 treatment at P5 there is a significant increase in cone sheath labelling by PNA and an increased expression of the three cone-specific markers GNAT2 and S- and M/L-opsin. These results indicate prolonged cone survival due to the restoration of RPE65 activity. Moreover, analysis of cone density in terms of RPE65 expression shows a transitional area where the therapeutic transgene is not detectable but cones are still spared.

### Therapeutic Window and Importance of the Vector Dose for *Rpe65* Gene Transfer

**Figure 5:**
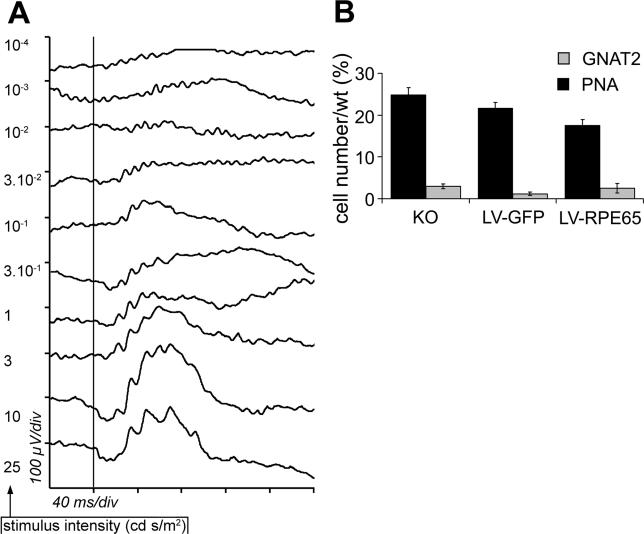
*Rpe65* Gene Transfer Performed at 1 mo Fails to Protect Cone Photoreceptors from Degeneration *Rpe65* ^−/−^ mice were treated by subretinal injection at age 1 mo with 20 ng of lentiviral vector. Treatment at this age with the LV-RPE65 vector restored ERG response with a threshold response of 3 × 10^−^^2^ cd s/m^2^ for the b-wave (A), whereas no improvement was observed in the LV-GFP treated group (unpublished data). (B) Counting of PNA-positive and GNAT2-positive cells revealed that *Rpe65* gene transfer performed at age 1 mo was unable to protect the cones from degeneration. Data are presented as mean ± SEM.

In utero rAAV-mediated *Rpe65* gene transfer in *Rpe65* ^−/−^ mice led to an improvement of the ERG [16], but the same type of vector failed to substantially restore retinal function when administered at 3 wk of age [17]. These studies indicate that there might be a small therapeutic window during which treatment outcome is optimal. To determine the ideal time point for lentivirus-mediated *Rpe65* gene transfer in *Rpe65* ^−/−^ mice, we injected lentiviral vectors in an additional group of mice at 1 mo of age. ERG responses were recorded 1 mo after injection (i.e., at 2 mo, the same age as for P5-treated animals). LV-RPE65 treatment at 1 mo increased retinal sensitivity (b-wave threshold) by 2 log units in comparison to LV-GFP-treated or untreated *Rpe65* ^−/−^ animals (Figure 5A). To identify whether this functional rescue reflects an improved cone survival, we studied cone rescue using PNA labelling and GNAT2 immunofluorescence after sacrifice of these mice at 3 mo of age. For both markers, we observed no improvement related to LV-RPE65 treatment (Figure 5B). In fact, the level of GNAT2-positive cells after LV-RPE65 treatment was 3% ± 1% of the wild-type GNAT2 amount, in the same range as for LV-GFP treated or untreated *Rpe65* ^−/−^ mice (Figure 5B). Thus, despite successful transduction of the RPE and improvement in the overall retinal sensitivity, cones were not rescued when mice were treated at 1 mo of age. These results clearly indicate that early cone loss [18,19] is irreversible and can be prevented only by early treatment (e.g., at P5, as described above).

**Figure 6:**
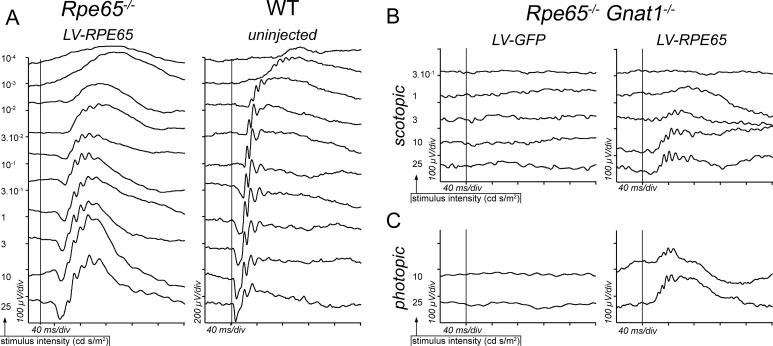
High Dose of LV-RPE65 Vector Restores a “Near Normal” ERG Response in *Rpe65* ^−/−^ Mice and Rescues the Activity of Cone Photoreceptors in *Rpe65* ^−/−^/*Gnat1a* ^−/−^ Mice (A) Scotopic ERG recorded at age 1 mo of a *Rpe65* ^−/−^ mouse treated at P5 with 180 ng of LV-RPE65 (left tracings) and of a *Rpe65* ^+/+^ untreated mouse (WT, right tracings). At this dose, LV-RPE65 treatment restored an ERG response similar to a wild-type response. For *Rpe65* ^−/−^ eyes showing the best restoration (*n* = 3), thresholds of both the a-wave and the b-wave reached normal levels with, respectively, 10−^1^ and 10−^3^ cd s/m^2^. (B and C) At P5 *Rpe65* ^−/−^/*Gnat1a* ^−/−^ mice received a bilateral intravitreal injection of the vector (LV-RPE65 or LV-GFP) at a dose of 180 ng of p24 capsid protein. 1 mo later, the ERG was recorded in scotopic (B) and in photopic conditions (C). Vertical bars indicate occurrence of the stimuli; x-axis: 40 ms/div; y-axis: 100 μV/div (left tracings), except right tracings in (A) (200 μV/div).

The vector dose is a second issue that is critical in view of a clinical application. To explore the importance of this parameter, we treated *Rpe65* ^−/−^ mice at P5 with a 9-fold higher dose of LV-RPE65 vector than in the previous experiments, i.e., 180 ng of p24 (corresponding to 5.5 × 10^5^ transducing units for the LV-GFP vector). In this case, we recorded the ERG after 1 mo, and we again observed heterogeneity in the improvement of the response after *Rpe65* gene transfer, but we also found animals with a “near-normal” ERG response characterized by a threshold of 10−^3^ cd s/m^2^, similar to wild-type animals (*n* = 3 eyes). In these animals, a-wave and b-wave amplitudes reached, respectively, 43.8% and 70% of the wild-type control means for the highest stimulus intensity (25 cd s/m^2^; Figure 6A, left tracings). Moreover, for the stimulus at which the ERG response reached a plateau (10 cd s/m^2^), we observed a higher b-wave amplitude after treatment with a high dose of the LV-RPE65 vector (119.2 ± 23 μV, *n* = 6) than after treatment with a low dose (69.6 ± 12.9 μV, *n* = 8, *p* = 0.032 determined by Student's t-test). ERG responses in animals treated with a high dose of LV-GFP vector (*n* = 6, unpublished data) were indistinguishable from untreated knockouts or animals treated with the low dose of LV-GFP vector (Figure 2A). This experiment suggests that the level of functional rescue in *Rpe65* ^−/−^ mice is improved by a higher dose of lentiviral vector.

### LV-RPE65 Treatment Restores Cone Function in RPE65-Deficient Mice

As rod and cone activity in *Rpe65* ^−/−^ mice cannot be readily distinguished with ERG recordings [6], we used an additional transgenic mouse line to prove that LV-RPE65 treatment rescues cone function. Mice lacking the rod transducin alpha subunit *(Gnat1a* ^−^ *^/^* ^−^ *)* [36] were crossed with *Rpe65* ^−/−^ mice to obtain *Rpe65* ^−/−^/*Gnat1a* ^−/−^ double knockout mice. In these mice, any retinal response to light after 11-*cis*-retinal supplied through *Rpe65* gene transfer cannot be derived from the rod transduction cascade, which is disrupted [32]. Similar to the previous experiment, we treated double knockout mice at P5 by intravitreal injection of LV-RPE65 (*n* = 5) or LV-GFP (*n* = 5) with 180 ng of p24 capsid protein, and we measured the ERG response after 1 mo. In the LV-GFP-treated group, we were not able to detect an ERG response to any stimulus intensity (Figure 6B, left tracings), similar to what is observed in untreated mice (unpublished data). In contrast, LV-RPE65 treatment of *Rpe65* ^−/−^ */Gnat1a* ^−/−^ mice restored an ERG response typical of cones, rising for the high-intensity stimuli and showing a bell-shaped curve (Figure 6B, right tracings). Furthermore, a response was also clearly detected when the ERG was recorded in photopic conditions (Figure 6C, right tracings). This, together with the fact that the rods of these mice are unable to function, demonstrates that lentivirus-mediated transfer of the *Rpe65* gene is able to rescue a functional response originating from the cones in a model of complete chromophore deprivation, the *Rpe65* ^−/−^ mouse.

## Discussion

We report a successful restoration of retinal function by lentivirus-mediated *Rpe65* gene transfer in an animal model of LCA. Furthermore, the rescue of the retinal function assessed by ERG is the best ever reported in the *Rpe65* ^−/−^ mouse model of LCA in terms of cone protection and visual sensitivity restoration [16,17], demonstrating the potential of this vector for a clinical application. In addition, we demonstrated that the use of the R0.8 promoter allows long-lasting expression of the *Rpe65* transgene mostly restricted to RPE cells. From a clinical perspective, preservation of cone vision is a critical endpoint—indeed is the final goal for human therapies. We demonstrate, using different markers specific for cones, that *Rpe65* gene transfer led to preservation of cone morphology and, in a transgenic line deficient for both RPE65 and the rod phototransduction cascade *(Gnat1a* ^−^ *^/^* ^^−^^ *)*, to restoration of cone function. The absence of ERG response in untreated or LV-GFP-treated *Rpe65* ^−^ *^/^* ^−^ */Gnat1a* ^−^ *^/^* ^−^ mice (Figure 6B and 6C), together with the fact that the residual response in *Rpe65* knockout animals originates from rods [6], indicate that the rod response is abolished in this mouse strain due to the knockout of the *Gnat1a* gene. The ERG response restoration after LV-RPE65 treatment of the double-knockout animals thus demonstrates that *Rpe65* gene transfer elicits a functional response from cone photoreceptors in the *Rpe65* ^−/−^ genetic background (Figure 6B and 6C).

As gene therapy trials for RPE65 deficiencies are on the verge of starting, it is important to accumulate data on different preclinical models using various vectors. The value of our results is reinforced by the fact that cone degeneration in *Rpe65* ^−/−^ mice starts just 2 wk after birth [18,19]. As a result, cone function is almost not recordable at P25 [18] and is completely lost by 4–5 wk of age [6]. Accordingly, we show here that LV-RPE65 prolongs cone survival for a protracted period (at least 4 mo, the latest time point studied) when administered at P5, but not at all when applied at 4–5 wk of age. These results are consistent with previous studies using rAAV in *Rpe65* ^−/−^ mice. Dejneka et al. found in utero treatment to be more efficient than treatment of adult mice (1 or 2.5 mo), but cone function and survival were not determined in this report [16]. In another study, by Lai et al., in mice injected at age 3 wk only 50% of the density of S-opsin labelling was restored at the site of injection at 8 mo [17]—consistent with our results showing that gene therapy given at 1 mo does not protect cones compared to that given at P5. Thus, the degeneration of cones beginning at age 2 wk [19] is irreversible, but can be prevented by early restoration of RPE65 activity (present study) or exogenous 11-*cis*-retinal [18]. Lentivirus-mediated gene transfer at P5 seems indeed optimal for cone protection in *Rpe65* ^−/−^ mice.

Consistent with the ability of lentiviral vectors to provide long-lasting expression in various cells and tissues in vitro as well as in vivo, and in the eye in particular [10,11], we observed long-term restoration of RPE function and preservation of cone photoreceptors, lasting at least 4 mo after gene transfer, the latest time point studied. Furthermore, phenotypic classification of LCA cases in humans placed RPE65 deficiencies in the rod-cone dystrophies group [8,37,38]. This classification suggests that, to act on cone function, which is the main therapeutic target to restore high-acuity vision for the patient, the intervention window will be proportionally even longer in human patients than in *Rpe65* ^−/−^ mice. Our results thus support a potential clinical application of *RPE65* gene transfer in humans.

For clinical trials it is also important to define accurately the vector dose that elicits a satisfactory therapeutic effect without triggering side effects. In the present study we show that a low virus dose restores a “near-normal” retinal sensitivity (Figure 2B) and completely blocks cone degeneration in the area expressing the therapeutic transgene, as well as in the vicinity of the treated area. Our study suggests that increasing the vector dose leads to an improvement of the ERG rescue (Figure 6A), but surgical limitations in mice resulted in the suggestion that increasing the vector dose leads to a higher level of expression in the transduced area rather to an expansion of the transduced area. The area of transgene expression might also be a critical parameter for effective ERG rescue: Above a certain threshold, retinal sensitivity is greatly enhanced (Figure 2C). Furthermore, the size of the transduced area is linearly correlated with the extent of cone survival area (Figure 3L), which exceeds the area of therapeutic transgene expression (Figure 3J). One explanation of this phenomenon could be heterogeneity in transduction efficiency within the injected area, the centre being highly transduced while the periphery expresses the transgene at a lower level (undetectable, but functionally relevant). Nevertheless, we never observed such heterogeneity after GFP or *Rpe65* gene transfer, and it has, to our knowledge, never been reported after subretinal injection of a gene transfer vector. Another possible explanation is that lateral diffusion of 11-*cis*-retinal promotes cone survival also around the actually transduced area. Local concentration (and thus diffusion) of the chromophore should increase with the size of the transduced area, explaining the linear relationship between the sizes of respectively transduced area and cone survival area (Figure 3L). This would imply that, in *Rpe65* ^−/−^ mice, cone survival depends more on the availability of a certain amount of the chromophore than on a restoration of the RPE65 function in the adjacent RPE cell. This hypothesis is reinforced by the observation that exogenously supplied chromophore protects cones until at least P25 in RPE65-deficient mice [18]. However, the exact mechanism underlying cone rescue following *Rpe65* gene transfer is still unknown, and we cannot exclude an indirect effect resulting, for example, from the participation of the Müller cells in the regeneration of cone pigments.

The strong correlation between the size of the actually transduced area and the size of the cone survival area is a critical point with regard to the treatment of patients. It may help to determine precisely the retinal region to be treated to reach a therapeutic level of 11-*cis*-retinal at the site of the fovea. To rescue high-acuity vision in human patients, it might thus be sufficient to target the vicinity of the fovea with a limited dose of vector. Such treatment would be achieved most efficiently by subretinal injection, a procedure already used for conventional drug delivery [39], and proposed for gene therapy clinical trials (US clinical trials 0410–677 and 0510–740; available at: http://www.gemcris.od.nih.gov/). Furthermore, from a clinical perspective, the existence of a transitional area where cones are rescued but transgene not expressed in RPE cells, is of prime importance to increase the safety of the procedure and optimize the restoration of the biological function. This could indeed allow for a therapeutic effect at the fovea without direct treatment of this zone, a crucial advantage in terms of surgical risk.

Using a rodent model of LCA, we show the potential of lentiviral vectors to efficiently restore a deficient function by specific gene transfer to the RPE. A recent study published by Yanez-Munoz et al*.* (2006) demonstrates that an integration-deficient lentiviral vector is as efficient as an integration-proficient vector for delivery of *Rpe65* in another model of LCA, the *Rpe65* ^rd12/rd12^ mouse [40]. These new in vivo data obtained with a vector minimizing mutagenesis risks by avoiding integration into the host genome reinforce our results that propose the lentiviral vector as an alternative for patient treatment. Moreover, it is important to note that stable and efficient retinal gene transfer by lentiviral vectors has already been documented in a nonhuman primate model [41], rendering this family of vectors very attractive for retinal applications targeting the retina. The next step toward a clinical application is to validate the use of classical lentiviral vectors, or of their integration-deficient version, using larger models such as RPE65-deficient dogs, and to analyze precisely the long-term efficiency of lentivirus-mediated RPE65 gene transfer in an eye similar in size to the human one. These studies should determine if the lentiviral vector could represent an alternative to rAAV for gene transfer into the RPE.

## Supporting Information

### Accession Numbers

The GenBank (http://www.ncbi.nlm.nih.gov/) accession numbers of the genes discussed in this paper are: enhanced green fluorescent protein (egfp) (U55761); human retinal pigment epithelium-specific 61 kDa protein (RPE65), promoter (AH006060); Mus musculus guanine nucleotide binding protein, alpha transducing 1 (Gnat1), mRNA (NM_008140); M. musculus retinal pigment epithelium 65 (Rpe65), mRNA (NM_029987); and woodchuck post-transcriptional regulatory element (WPRE, nucleotides 1093–1684) (J04514).

## Abbreviations

cd: candela(s)
PNA: peanut agglutinin lectin
rAAV: recombinant adeno-associated virus
RPE: retinal pigment epithelium
P[number]: postnatal day [number]
SEM: standard error of the mean
